# Explainable AI for CNN-based prostate tumor segmentation in multi-parametric MRI correlated to whole mount histopathology

**DOI:** 10.1186/s13014-022-02035-0

**Published:** 2022-04-02

**Authors:** Deepa Darshini Gunashekar, Lars Bielak, Leonard Hägele, Benedict Oerther, Matthias Benndorf, Anca-L. Grosu, Thomas Brox, Constantinos Zamboglou, Michael Bock

**Affiliations:** 1 Department of Radiology, Medical Physics, Medical Center University of Freiburg, Faculty of Medicine, University of Freiburg, Freiburg, Germany; 2 German Cancer Consortium (DKTK), Partner Site Freiburg, Freiburg, Germany; 3 Department of Radiology, Medical Center University of Freiburg, Faculty of Medicine, University of Freiburg, Freiburg, Germany; 4 Department of Computer Science, University of Freiburg, Freiburg, Germany

**Keywords:** Convolutional neural network, Automatic prostate tumor segmentation, Histological validation

## Abstract

Automatic prostate tumor segmentation is often unable to identify the lesion even if multi-parametric MRI data is used as input, and the segmentation output is difficult to verify due to the lack of clinically established ground truth images. In this work we use an explainable deep learning model to interpret the predictions of a convolutional neural network (CNN) for prostate tumor segmentation. The CNN uses a U-Net architecture which was trained on multi-parametric MRI data from 122 patients to automatically segment the prostate gland and prostate tumor lesions. In addition, co-registered ground truth data from whole mount histopathology images were available in 15 patients that were used as a test set during CNN testing. To be able to interpret the segmentation results of the CNN, heat maps were generated using the Gradient Weighted Class Activation Map (Grad-CAM) method. The CNN achieved a mean Dice Sorensen Coefficient 0.62 and 0.31 for the prostate gland and the tumor lesions -with the radiologist drawn ground truth and 0.32 with whole-mount histology ground truth for tumor lesions. Dice Sorensen Coefficient between CNN predictions and manual segmentations from MRI and histology data were not significantly different. In the prostate the Grad-CAM heat maps could differentiate between tumor and healthy prostate tissue, which indicates that the image information in the tumor was essential for the CNN segmentation.

## Introduction

Prostate carcinoma (PCa) is the most common malignant tumor in men in Europe and in the United States of America. Early detection of PCa is important to select the appropriate type of cancer treatment. Elevated levels of the prostate specific antigen (PSA) combined with a digital rectal exam are used as early markers for a further evaluation and decision-making. Multiparametric magnetic resonance imaging (mpMRI) is currently used as a standard protocol for diagnosing, staging, and definitive management of PCa in clinical practice [1]. mpMRI demonstrated excellent sensitivity in the detection of PCa by providing high soft-tissue contrast and differentiation of internal structures and surrounding tissues of the prostate [2–4]. Due to the complexity associated with the location and size of the prostate gland, manual and accurate delineation of PCa from healthy tissue is time consuming and susceptible to high inter- and intra-observer variability [5–8]. Hence, there is a need for automated algorithms for robust segmentation of clinically significant PCa with a biopsy Gleason score of 6 and above.

Algorithms based on convolutional neural networks (CNNs) have shown promising results for PG segmentation of the whole PG and the PG zones [9–20]. Even though CNNs perform well in PCa segmentation [16, 21–24], the training of the CNN remains challenging due to the absence of verified ground truth image data, as biopsy data is only available at a limited number of locations in the gland. Another problem of CNNs has been attributed to the intransparency associated with the way in which a CNN comes to a decision, which does not foster trust and acceptance amongst the end users. Hence, there is a need for explainable models that quantify why certain predictions were made [25].

Recently, the gradient-weighted class activation mapping (Grad-CAM) method was proposed by Selvaraju et al. [26] for visualizing the important regions for decision making. The Grad-CAM method leverages the spatial information preserved through convolutional layers to understand which parts of an input image were important for a classification decision. The output of the Grad-CAM method is a class discriminative localization map (heat map) which highlights the most salient/most important pixels of a particular class. Grad-CAM has been applied in numerous research areas and is particularly popular in the medical domain. Kim et al. [27] used the Grad-CAM method to classify various medical imaging modalities. Yang et al. [28] extended the Grad-CAM method to generate 3D heat maps for the classification of Alzheimer’s disease. However, these methods are widely used for the interpretation of classification decisions [29], but have rarely been applied for segmentation tasks. Hoyer and Khoreva [30] proposed a method for the visual explanation of semantic segmentation CNNs based on perturbation analysis, with the assumption that co-occurrences of some classes are important for the task of segmentation, thus focusing on identification of contextual biases. Vinogradova et al. [31] proposed SEG-GRAD-CAM, an extension of Grad-CAM for semantic segmentation, for generating heat maps to explain the importance of individual pixels or regions in the input image for semantic segmentation. Couteaux et al. [32] proposed a method inspired by Deep Dream [33], for the interpretation of segmentation networks to generate and analyze false positives by maximizing the activations of the neuron using a gradient ascent method to provide insights on the sensitivity and robustness of the trained network to specific high-level features. However, the method is yet to be tested on architectures such as U-net [34], DeepLab [35] or PSPNet [36].

In this study, we use a U-net type CNN for the automated segmentation of two structures: the prostate gland (PG) and the PCa. To validate the CNN for the task of PCa segmentation against an established ground truth, whole mount histopathology slices from prostatectomy patients are used that are co-registered with mpMRI images by using an established framework for imaging/histopathology registration [37]. As segmentation is essentially a localization followed by a classification of a group of pixels belonging to a target class. Here, we generalize the 3D-Grad-CAM and SEG-Grad-CAM segmentation method proposed in [28–31]. To interpret how the CNNs organize themselves internally for PG and PCa segmentation, we provide explanations in the form of heatmaps.

## Materials and methods

### Clinical data

In this study, mpMRI data from histologically confirmed primary PCa patients was used (histopathological samples obtained by biopsy). The data consists of two groups, an internal data set (n = 15/122, with/without whole mount histology data). Examinations were acquired between 2008 and 2019 on clinical 1.5 T (Avanto, Aera & Symphony, Siemens, Erlangen, Germany) and 3 T (Tim TRIO, Siemens, Erlangen, Germany) MRI systems. Images were acquired with surface phased array (body matrix) coils in combination with integrated spine array coils – note, that no endorectal RF coil was used. Tables 1, 2 gives an overview of the sequence parameters used for imaging in 3 T and 1.5 T systems respectively. The study was approved by the institutional ethics review board (Proposal Nr.476/14 & 476/19) and patients gave written informed consent.

**Table 1:** MRI sequence parameter for 3 T

Sequence	TR (ms)	TE (ms)	Resolution (mm^3^)	Slice thickness (mm)	Slice gap (mm)	flip angle	FOV (mm)	Matrix	b values (s/mm^2^)
T2-TSE	5500	103–108	0.78 × 0.78 × 3	3		150°	150 × 150	192 × 192	
DWI-EPI	3500	73	1.56 × 1.56 × 3	3	0	90°	250 × 250	160 × 160	50, 400, 800
DCE-MRI	5.13	2.45	1.35 × 1.35 × 3	3		12°	260 × 260	192 × 162

**Table 2:** MRI sequence parameter for 1.5 T

Sequence	TR (ms)	TE (ms)	Resolution (mm^3^)	Slice thickness (mm)	Slice gap (mm)	Flip angle	FOV (mm)	Matrix	b values (s/mm^2^)
T2-TSE	8650–9400	111–119	0.78 × 0.78 × 3	3		150°	150 × 150	192 × 192	
DWI-EPI	2800 –3840	61–87	1.56 × 1.56 × 3–2.5 × 2.1 × 6	3–6	0–0.5	12°	300 × 300–400 × 338	192 × 162 160 × 160	0, 100, 400, 800 or 0, 250, 500, 800
DCE	4.65–4.1	1.58–1.6	1.35 × 1.35 × 2 1.04 × 1.04 × 3	2–3		12°–15°	260 × 260 400 × 387	192 × 192–384 × 372	

Imaging protocol was as follows: T2-weighted turbo spin echo (TSE) images in transverse, sagittal and coronal orientation, DWI with an echo planar imaging sequence in transverse orientation. DWI data were acquired with b-values of [0, 100, 400, 800] s/mm^2^ or [0, 250 500, 800] s/mm^2^ for 1.5 T, and [50, 400, 800] s/mm^2^ for 3 T. With the DWI data, a synthetic high b-value image was calculated for each patient. The $\mathrm{ADC}$ and ${S}_{0}$ were fitted pixel wise according to Eq. (1)

1 $$S= {S}_{0}{e}^{-b \mathrm{ADC}} .$$

Using these fitted values, a synthetic diffusion-weighted image with a b-value of 1400 s/mm^2^ was calculated for all the patients.

The protocol included additional dynamic contrast enhanced imaging, which were not part of the CNN-based analysis.

**Fig. 1:**
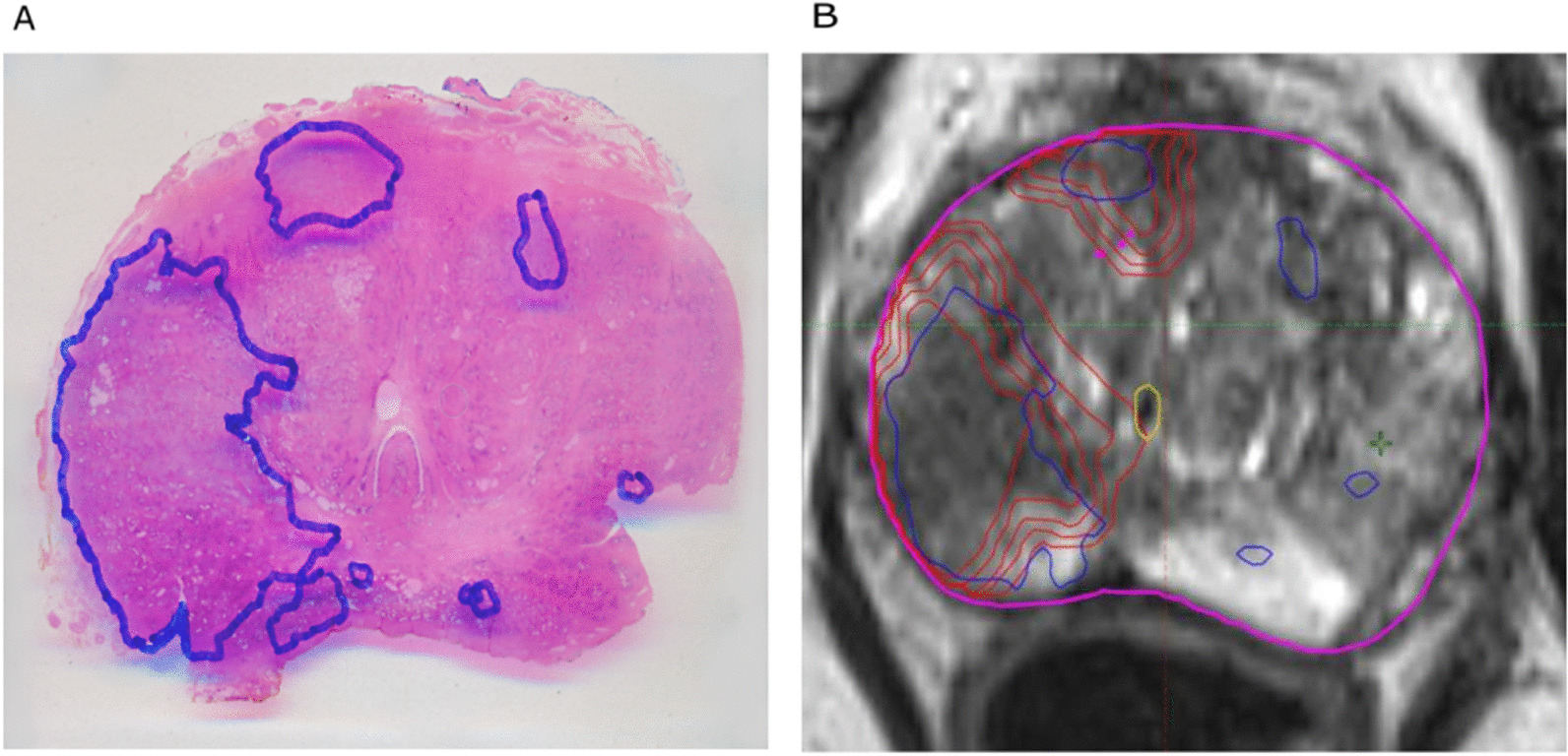
A sample histology reference projected on the MRI sequence: (**A**) Hematoxylin and eosin whole-mount prostate slide with marked PCa lesion. (**B**) Registered histopathology slice blue = PCa- Histo, red = PCa-Rad with 1 mm isotropic expansion

Patient data was separated into a training cohort and a test group: The training cohort consists of a large irradiation and prostatectomy group (*n*_*irr*_ = 122), and the test cohort consists only of a prostatectomy group (*n*_*prost*_ = 15) from which whole organ histopathology slices were available. The mpMRI data to train the CNN contained T2 weighted images and apparent diffusion coefficient (ADC) maps together with synthetic high b-value images (b = 1400 s/mm^2^). For all 137 in house mpMRI images (*n*_*irr*_ and *n*_*prost*_), the entire PG (PG-Rad), and PCa (PCa-Rad) within the prostate were contoured by two experienced radio-oncologists. As in [37, 38], PCa (PCa-Histo) tissues in the whole mount histology data from the test cohort were stained with hematoxylin and eosin. Tumor contours were then delineated by experts and digitized. These whole mount histology slices were intermediately registered with the corresponding T2 weighted ex vivo MRI using MITK software (MITK Workbench 2015.5.2). The histopathology slices and ex-vivo MRI images were registered using anatomical landmarks, by prioritizing the agreement between the prostate capsule contours, the urethra and cysts. Automatic interpolation was performed to generate 3D volumes. The ex-vivo MRI images along with the histology-based tumor contours (PCa-Histo) were imported into the radiation therapy planning system Eclipse v15.1 software (Varian Medical Systems, USA). Here, a careful manual co-registration of the ex-vivo MRI (PCa-Histo) and in-vivo MRI (PCa-Rad) was performed using anatomical landmarks, allowing for non-rigid deformation. All contours (PCa-Histo, PCa-Rad) were later transferred to the corresponding in vivo MRI image (cf. Fig. 1).

For data preprocessing, the mpMRI sequences were cropped to a smaller FOV around the prostate gland and then registered and interpolated to an in-plane resolution of 0.78 × 0.78 × 3 mm^3^. Due to the large sizes of the image volumes which would result in very long computation times, calculations were performed on patches of size 64 × 64 × 16 that were chosen randomly with respect to the center location of the original image. The probability of the center pixel to be of the class background (BG), PCa or PG was set to 33% to account for class imbalance and a chance of 70% for a random 2D-rotation in the axial plane was added for data augmentation.

### Convolutional neural network

A patch-based 3D CNN of the U-net architecture [34] was trained for the automatic segmentation of PCa and PG. The network was implemented in MATLAB® (2020a, MathWorks, Inc., Natick/MA) using the deep learning toolbox. The CNN consists of 3 encoder blocks for down sampling steps with max-pooling, 3 decoder blocks for up sampling steps with transposed convolution layers (kernel size:2 × 2 × 2, stride:2, padding:1) and skip connections by concatenation. The convolution blocks consist of 3 × 3 × 3 convolutions with stride and padding of 1, followed by batch normalization and Rectified Linear Unit activation (ReLU), except for the last convolution where 1 × 1 × 1 convolution without padding, batch normalization and softmax activation function were used.

The CNN was trained using optimal parameters learning rate 0.001, patch size 64 × 64 × 16 obtained by a Bayesian optimization scheme to maximize the segmentation performance within 150 epochs on an NVIDIA RTX2080 GPU. During the CNN testing phase, the mpMRI data from the test cohort (prostatectomy group) was used to evaluate the network prediction. The resulting segmentation is evaluated by comparing the Dice Sorensen Coefficient (DSC) with the ground truth.

### 3D: grad-CAM for segmentation

**Fig. 2:**
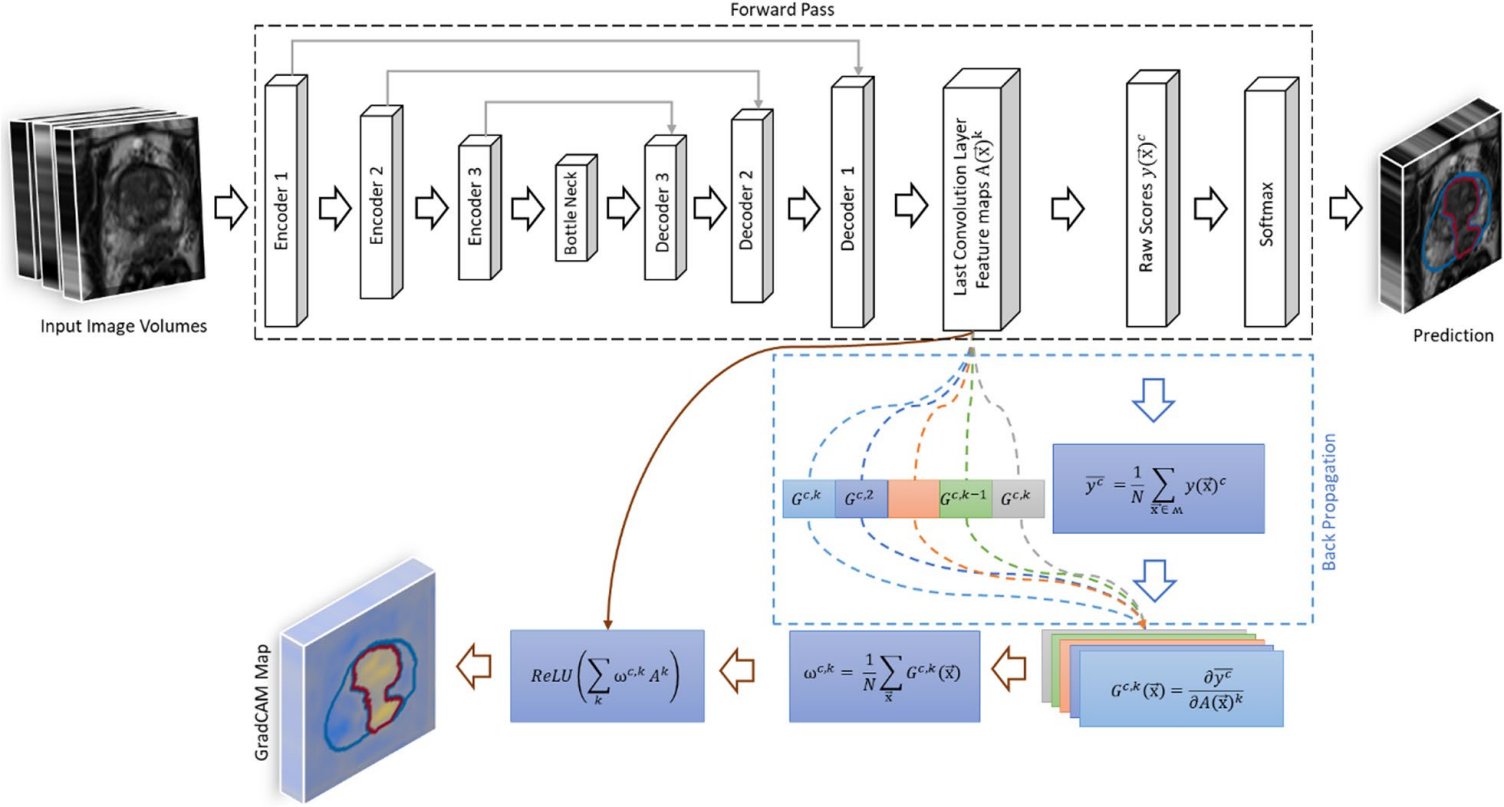
Overview 3D – Grad-CAM method for segmentation. Black arrows indicate forward pass, the blue arrows indicate the back propagation & the brown arrows indicate the further steps for generating the Grad-CAM maps

The Grad-CAM method proposed by [26] is generalized to be applied to a pre-trained CNN with fixed learned weights in a segmentation task. Yang et al. [28] extended the Grad-CAM method to 3D-Grad-CAM. A schematic of the 3D-Grad-CAM is shown in Fig. 2. Here, for understandability, let ${\{{\mathrm{A}(\overrightarrow{\mathrm{x}})}^{\mathrm{k}}\}}_{\mathrm{k}=1}^{\mathrm{K}}$ be a set of selected feature maps of interest from $\mathrm{K}$ kernels of the last convolutional layer of the CNN, and ${\mathrm{y}(\overrightarrow{\mathrm{x}})}^{c}$ be the raw score of the CNN for a chosen class $c$ before softmax activation. The Grad-CAM method first computes the gradients ${G}^{c,k}(\overrightarrow{\mathrm{x}})$ of class scores ${y(\overrightarrow{\mathrm{x}})}^{c}$ with respect to all *N* voxels for each feature map ${A(\overrightarrow{\mathrm{x}})}^{k}$ of the convolutional layer:

2 $${G}^{c,k}\left(\overrightarrow{\mathrm{x}}\right)=\frac{ \partial {\mathrm{y}\left(\overrightarrow{\mathrm{x}}\right)}^{c}}{\partial {A\left(\overrightarrow{\mathrm{x}}\right)}^{k}} .$$

These gradients are then globally average-pooled in all three spatial dimensions to obtain neuron importance weight ${\upomega }^{c,k}$:

3 $${\upomega }^{c,k}= \frac{1}{N}\sum_{\overrightarrow{\mathrm{x}}\boldsymbol{ }\boldsymbol{ }}{G}^{c,k}\left(\overrightarrow{\mathrm{x}}\right) .$$

Then, a heat map ${H(\overrightarrow{\mathrm{x}})}^{c}$ is computed by summation of the feature maps ${A(\overrightarrow{\mathrm{x}})}^{k}$ multiplied by their corresponding weight ${\upomega }^{c,k}$ and subsequent ReLU activation to suppress negative contributions:

4 $${H(\overrightarrow{\mathrm{x}})}^{c} =ReLU\left(\sum_{k}{\upomega }^{c,k}{A}^{k}\right)$$

Segmentation is essentially a classification of each voxel in the input image $\mathrm{I}(\overrightarrow{\mathrm{x}})$ to a category of target labels ${\mathrm{y}(\overrightarrow{\mathrm{x}})}^{\mathrm{c}}$. Thus, from the method proposed in [39], we generalize the 3D Grad-CAM method for segmentation, by averaging the class score ${y(\overrightarrow{\mathrm{x}})}^{c}$ for a set of voxels in the output segmentation mask ʍ as in Eq. 55

The algorithm was implemented using the dlfeval function from the Deep Learning tool box in MATLAB® (2020a, MathWorks, Inc., Natick/MA).

### Evaluation of heat maps

The quality of the generated heat maps for its localization ability is evaluated using the intersection over Union (IOU) metric. For this, as proposed in [40], the generated heat maps for the test images are min–max normalized. Then, they are thresholded at different intensity values δ to generate binary masks (${L}^{c}$) by converting the intensity values above δ to one and below δ to zero. Finally, we calculate the IOU (${Loc}^{c}(\delta )$) between the ground truth segmented label (${y}_{Gt}^{c}$) and the binary map (${L}^{c}$) for a class c thresholded at value δ for the test image $\mathrm{I}(\overrightarrow{\mathrm{x}})$ as,

6 $${Loc}^{c}\left(\delta \right)=\frac{{L}^{c}(\delta )\cap {y}_{Gt}^{c}}{{L}^{c}(\delta )\cup {y}_{Gt}^{c}}$$

A higher value of, ${Loc}^{c}(\delta )$ is indicative of a better localization of the heat map for the target class.

For the sanity check, the model randomization test and the independent cascaded randomization test proposed in [41] is used to study the sensitivity of the heat maps with the learned parameters of the CNN. For the model randomization test, we generate heat maps from an untrained U-Net model with random weights and bias, which are then compared to the heat maps from the trained network. For the independent cascaded randomization test, the weights of the convolutional layers in the decoder and encoder blocks are independently randomized from top to the bottom of the network in a cascading manner and the heat maps are generated. Finally, we compare the mutual information and SSIM between the heat maps generated from the learned model with fixed weights, model randomization test and independent cascaded randomization test.

## Results

**Fig. 3:**
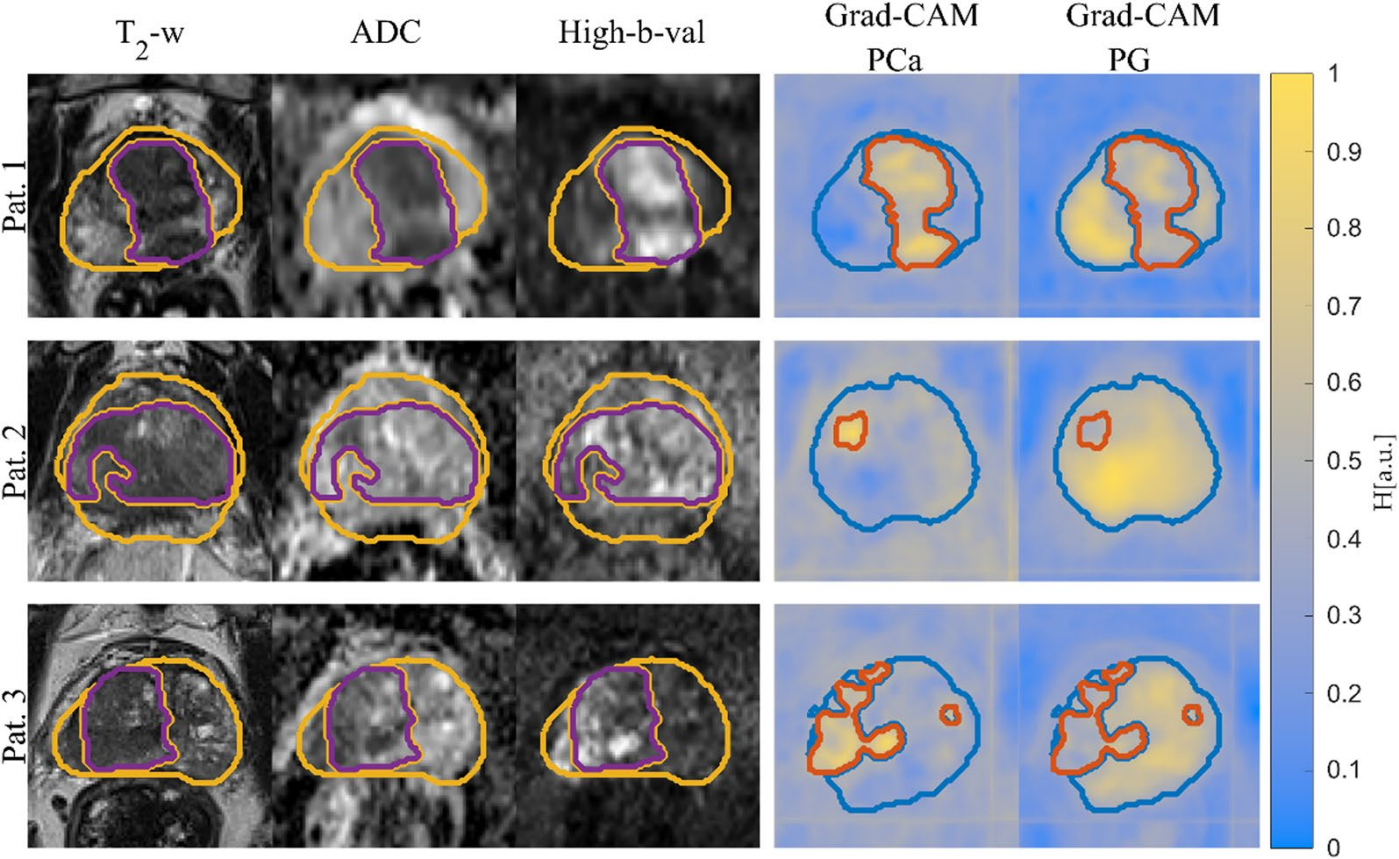
Segmentation of PG and PCa for test patient 1 -3 with the corresponding input mpMRI sequences and ground truth labels PG (yellow) & PCa (purple). The corresponding Grad-CAM maps are overlaid with the network predicted segmentation for PG (blue) & PCa (orange)

Figure 3 shows input sequences, ground truth, predicted segmentation overlaid on the Grad-CAM map for test patients 1 to 3 from the test cohort for PCa & PG. The overlay highlights the regions with high activations, which the CNN deemed important for the predicted segmentation. The DSC for PCa (CNN-Histo) is 0.48, 0.64, and 0.10, for PCa (CNN-Rad) is 0.51, 0.80 and 0.13 and for PG (CNN-Rad) it is 0.49, 0.67 and 0.51, respectively.

**Fig. 4:**
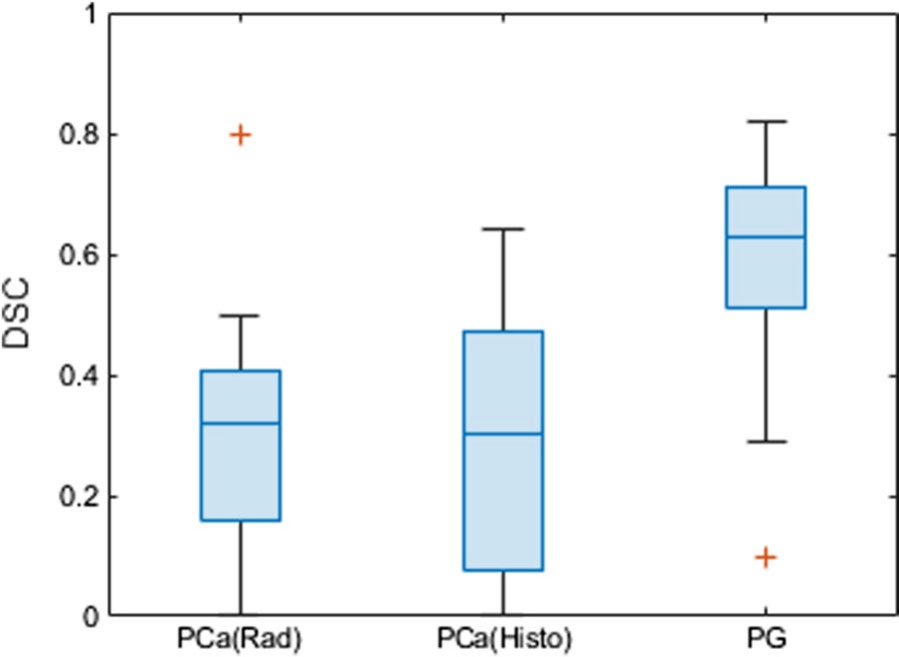
DSC for Test cohort (n = 15). The red lines in the plot show the median DSC value for the classes PCa and PG (CNN-Rad = CNN Predicted segmentation with Radiologist drawn cantors & CNN-Histo = CNN Predicted segmentation with whole mount histology cantors). The upper and lower bounds of the blue box indicate the 25th and 75th percentiles, respectively

**Fig. 5:**
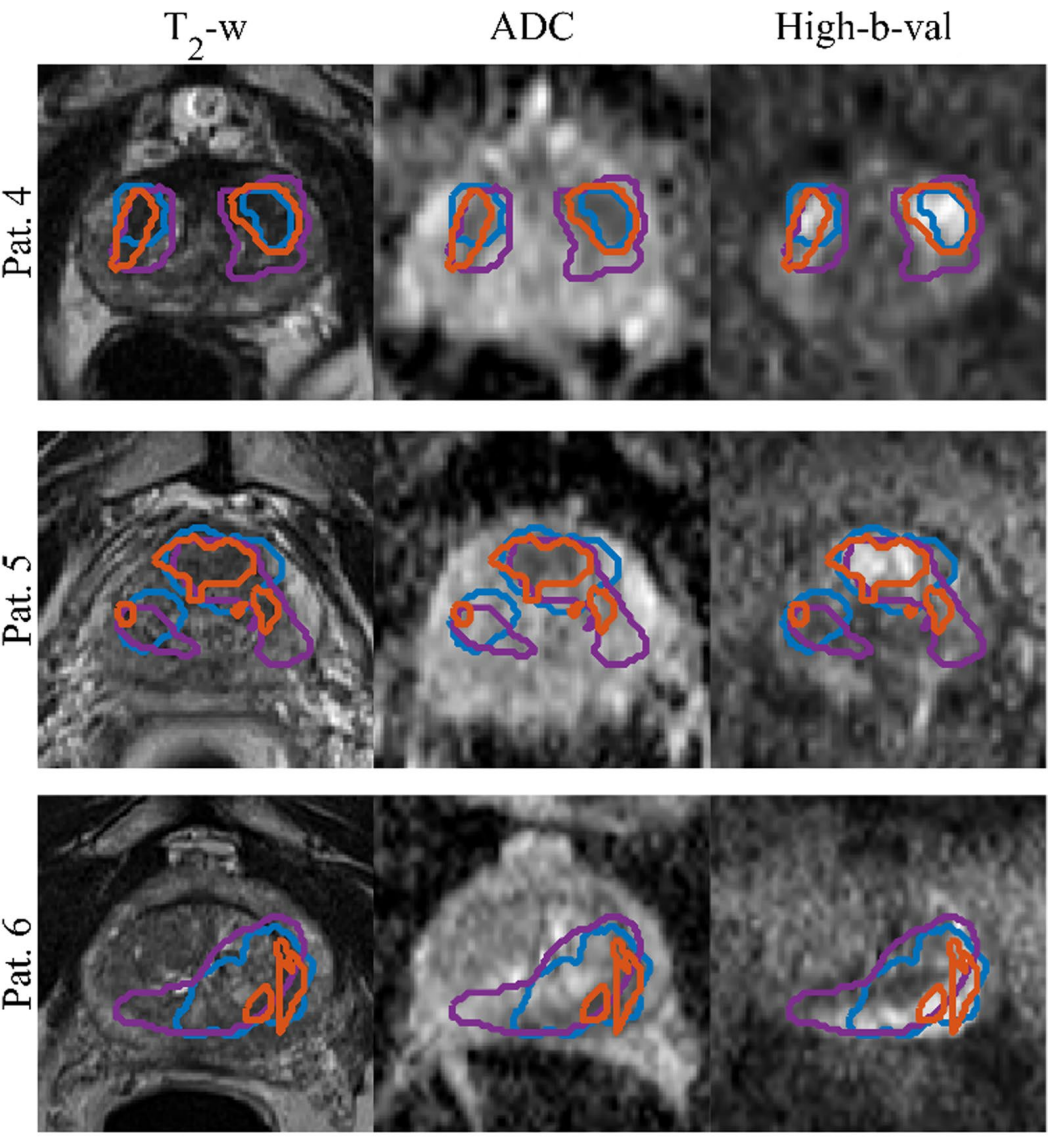
Segmentations of GTV overlaid on the input image sequences for patients from the test set. Ground truth segmentations PCa-Histo (purple), PCa-Rad (blue) and the predicted segmentation PCa-CNN (orange)

The mean, standard deviation and the median DSC between the CNN-predicted segmentation and the ground truth across the test cohort was 0.31, 0.21 and 0.37 (range: 0.64–0) for PCa (CNN-Histo), 0.32, 0.20 and 0.33 (range: 0.80–0) for PCa (CNN-Rad) and 0.62, 0.15, and 0.64 (range: 0.81 – 0.27) for PG (CNN-Rad) (Fig. 4) respectively. Figure 5 shows the CNN-predicted segmentation in comparison with the two ground truths PCa –Histo and PCa-Rad for test patients 4 and 5. The DSC for PCa (CNN-Histo) is 0.49, 0.44, and 0.07, for PCa (CNN-Rad) is 0.32, 0.39 and 0.15, for PG (CNN-Rad) it is 0.67, 0.60 and 0.23, respectively.

**Fig. 6:**
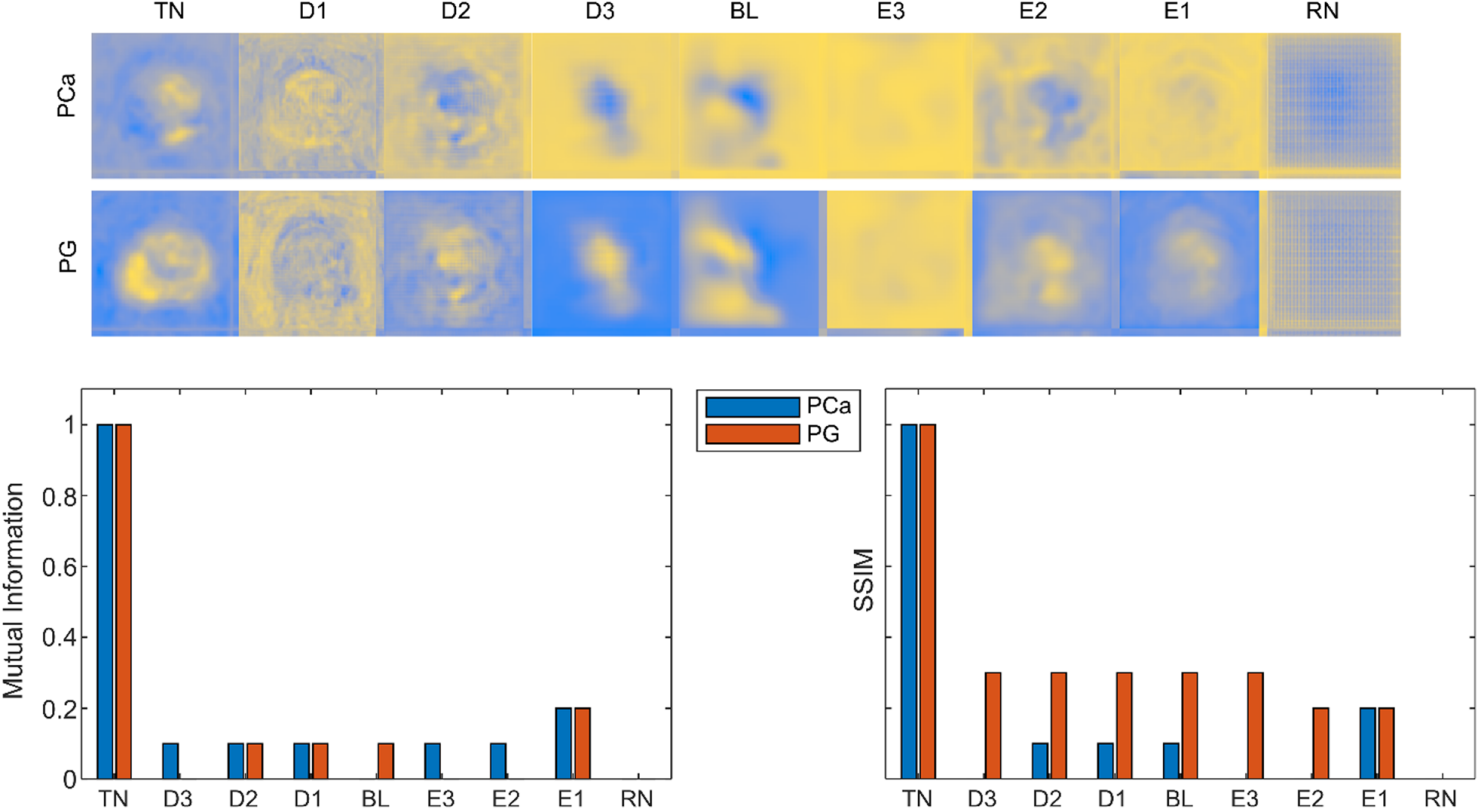
Cascaded randomization test. The first column shows the original Grad-CAM map for tumor (PCa) and Prostate (PG), followed by the Grad-CAM maps generated after randomizing the weights of the respective convolutional layers. Here TN is the trained Network, BL is the bottleneck layer, D1, D2, D3, E1, E2, E3 are the corresponding decoder and encoder blocks of the U-net, and RN is the network with random weights only

The mean and the standard deviation of the IOU per class (PCa & PG) for different δ values across the test set is presented in the Table 3. Figure 6 shows the heat maps generated from the cascaded randomization test for test patient 1, the mutual information and the SSIM values calculated between the heat maps from the trained model and the model randomization test, and the independent cascaded randomization test. MI and SSIM decreases from 1 to 0 between the heat maps generated from the trained network with fixed learned weights and from an untrained model with random weights.

**Table 3:** IIOU Results for class conditional localization of PCa and PG on the test set (higher is better)

	PCa	PG
$Mean {Loc}^{c}(\delta=0)$	0.03	0.16
$Mean {Loc}^{c}(\delta =0.25$)	0.04	0.21
$Mean {Loc}^{c}(\delta =0.50)$	0.15	0.47

The IOU improves with greater values of δ

However, for the cascaded randomization test, independent randomization of the learned weights decreased MI and SSIM to 0.26 ± 0.01 and 0.22 ± 0.01 across all test patients. This effect can also be observed in the heat maps of the cascaded randomization test: at all stages some structure of the original input image is preserved (Fig. 6).

## Discussion

In this study, mpMRI was combined with corresponding whole mount histopathology slices to evaluate the overall quality and the plausibility of a CNN for PCa segmentation. With an average segmentation performance of 0.31 ± 0.21 for PCa(CNN-Histo) and 0.32 ± 0.20 for PCa(CNN-Rad), the segmentation quality of the CNN was relatively low, but comparable to the value of 0.35 found in similar studies [5, 21]. A unique feature of this study is that the result was obtained by comparison against registered histopathology slices from the resected prostate which is considered to be the best available ground truth. The network, however, was not trained on histopathology, but on tumor contours drawn in the MRI according to the PI-RADS classification system [42], which is the established radiology standard for prostate cancer MRI. Recently, it was shown that PI-RADS-defined tumor contours underestimate the true tumor volumes [43] – thus, CNNs using this information might inherently also lead to a systematic underestimation of tumor volumes.

Inter-observer variability with a mean DSC in the range of 0.48–0.52 has been reported [5–8] and this low to intermediate agreement is expected to set an upper limit to the achievable prediction quality of the network. In this work, the manual MRI-based consensus segmentations from two experts (PCa-Rad), and the histopathological ground truth (PCa-Histo) were compared with the CNN predicted segmentations (PCa-CNN). The network predictions agree very well with those from PCa-Histo and PCa-Rad, but in 5 cases the prediction quality is low (DSC = [0, 0, 0.1, 0.1, 0.1]). A detailed analysis of the mpMRI data of these patients showed that one patient had residual bleeding post biopsy. The other 4 patients had no bleeding, or no inflammation, but showed a pronounced benign prostate hyperplasia (BPH) which could have influenced the predictions of the CNN. Nevertheless, the results indicate that the network learned to discriminate between healthy and diseased tissue rather than reproducing contours defined by a radio-oncology expert. This information could be useful in developing a segmentation-based detection and grading system similar to the work proposed in [44, 45].

Heat maps were generated based on the Grad-CAM method to interpret the recognition and localization capability the CNN. The heat maps and ground truth show the highest IOU at a threshold value $\delta$ of 0.5, revealing a strong correlation between them. The heat maps localize the respective classes correctly, without the sign of a “clever-Hans” artifact [46]. This analysis is a fundamental step before the application to new data (e.g., other tumor entities) to prevent false classification of pixels due to artifacts that might be inherent to the images or the algorithm. The segmentation performance is expected to increase with increasing localization of the tumor, i.e., a better delineation in the heat maps. This distinction has to be made, because the Grad-CAM heat maps describe the localization of the CNN attention, in contrast to the CNN segmentation, which only considers the resulting class activation that can originate from anywhere within the receptive field of the network.

The model randomization test was performed as a sanity check to eliminate systematic errors in the network. The cascaded randomization test however can be used to track information content within the network. MI and SSIM between the randomized and the trained networks should amount to 0, however in this study the MI varies between 0 to 0.2, and the SSIM between 0 to 0.4. Here, the variations in MI and SSIM at the decoder blocks might be caused by the flow of information via skip connections. Similarly, the variations at the encoder blocks could indicate the flow of information from the convolutional layers of the initial encoder blocks with learned weights [47]. Even with partly randomized weights, the network is able to recognize distinct structures in the image, indicating robustness against small errors. This kind of robustness, or resilience, is a vital part of any system that is supposed to be used in a clinical environment, and thus needs to be evaluated using established methods. As shown, the cascaded randomization test proves to be a valuable tool for this task.

A limitation of the study is the heterogeneity in the mpMRI images as the images were acquired from MRI systems with two different field strengths. To compensate for different diffusion weightings (b-values (1.5 T: b = [0, 100, 400, 800] s/mm2 or [0,250,400,800] s/mm2 and 3 T: [50, 400, 800] s/mm2), we calculated synthetic DWI images with b = 1400 s/mm2. For the T2-weighted image, no homogenization method was used, as the tissue T1 and T2 values are field strength-dependent. However, we expect the image contrast in the T2-weighted TSE images from the 1.5 T and 3 T systems to be similar, because the 1.5 T and 3 T T2-values in a wide range of human tissue are very similar [48] and repetition times greater than 5500 ms were used to minimize the T1 contrast. Another limitation is the low number of data available for testing (*n*_*prost*_ = 15 (~ 12%) of the training set), in comparison to other studies for which hundreds of test cases were available.

The use of whole-mount histology data as true ground truth is promising, however there are challenges associated with using this data, as the dataset consist of patients with intermediate and high risk PCa category that undergo prostatectomy and does not include patients from the low-risk category. In addition, the prostate gland tends to deform nonlinearly after prostatectomy, or during formalin embedding and cutting. These deformations could be challenging during the registration of the whole-mount histology data with mpMRI images, leading to bias in the ground truth.

## Conclusion

In this work, we demonstrated the application and benefits of explainable AI tools to tumor segmentation networks for PCa segmentation. A U-net CNN trained on expert contours was evaluated against histopathological ground truth. Although the segmentation performance can still be increased, the network passed all sanity checks and could be used to provide an initial tumor contour for further refinement by an expert. The evaluation by the Grad-CAM method further helps to explain the segmentation results thus fostering trust in the CNN prediction.

## Data Availability

The datasets used and/or analyzed during the current study are available from the corresponding author on reasonable request.
